# Sleep in Different Types of Dizziness

**DOI:** 10.1002/brb3.71863

**Published:** 2026-10-20

**Authors:** Lucia Joffily, Marco Antônio Sales Dantas de Lima, John Fontenele Araújo, Diego Kaski

**Affiliations:** ^1^ Department of Neurology Universidade Federal Do Rio de Janeiro (UFRJ) Rio de Janeiro Brazil; ^2^ SENSE Research Unit, Department of Clinical and Movement Neurosciences, Institute of Neurology University College London London UK; ^3^ Department of Otolaryngology Universidade Federal Do Estado Do Rio de Janeiro (UNIRIO) Rio de Janeiro Brazil; ^4^ Laboratório De Neurociência e Psicologia Social ‐ LaNPSo Universidade Federal do Delta do, Parnaíba – UFDPar, Rio de Janeiro, Brazil

**Keywords:** dizziness, sleep apnea, actigraphy, vestibular migraine, persistent postural‐perceptual dizziness, social jetlag

## Abstract

**Objective**: Sleep disturbances and dizziness are frequently comorbid, yet the specific sleep profiles across different vestibular disorders remain poorly characterized. This study aimed to investigate distinct sleep signatures in patients with benign paroxysmal positional vertigo (BPPV), vestibular migraine (VM), and persistent postural‐perceptual dizziness (PPPD) compared to healthy controls. **Methods**: We conducted a cross‐sectional study with 90 participants: 24 healthy controls, 21 with BPPV, 23 with PPPD, and 22 with VM. Sleep was assessed using a multimodal approach: 14‐day actigraphy, a single‐night home sleep apnea test (Watch‐PAT), and validated questionnaires, including the Pittsburgh Sleep Quality Index (PSQI), Dizziness Handicap Inventory (DHI), and Beck Anxiety/Depression Inventories. **Results**: All dizziness groups reported significantly worse subjective sleep quality and higher psychological burden than controls. However, each disorder presented a unique objective signature: **BPPV**: Characterized by objective sleep fragmentation and a markedly higher Apnea‐Hypopnea Index (AHI: 31.5) and Oxygen Desaturation Index (ODI: 16). These patients also showed increased nocturnal restlessness (L5). **PPPD**: Demonstrated a “sleep paradox” with the highest objective sleep efficiency but the poorest subjective perception (PSQI: 11.0). Patients showed reduced daytime activity (M10) and rigid sleep routines with virtually no weekend‐weekday get‐up time difference. **VM**: Distinguished by the shortest total sleep time and pronounced, though not statistically significant, “social jetlag”, reflected by a 120‐min discrepancy between weekend and weekday get‐up times. **Conclusion**: Dizziness is not a homogeneous entity regarding sleep. The distinct signatures found suggest that clinical management should be tailored: screening for sleep apnea in BPPV, addressing the objective‐subjective disconnect in PPPD via cognitive‐behavioral approaches, and managing sleep deprivation and circadian regularity in VM.

## Graphical abstract

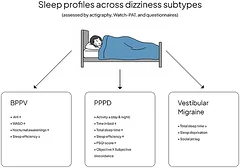

## 1 Introduction

Sleep‐related conditions are a diverse and common group of disorders that negatively affect individual well‐being and impose substantial socioeconomic costs (Abad and Guilleminault 2003). For men, the prevalence of sleep disorders ranges from 13.7% (ages 70–74 years) to 18.1% (ages 18–24 years) and for women, from 17.7% (80 years or older) to 25.1% (ages 18–24 years) (Grandner 2020). Insomnia and obstructive sleep apnoea (OSA) are the most common sleep‐related conditions and often coexist with physical and psychiatric disorders, including dizziness (Yu et al. 2025; Schifino et al. 2025).

Dizziness affects up to 30% of the general population and represents a leading cause of referral to otolaryngology and neurology services worldwide (Neuhauser 2016; Bhattacharyya et al. 2017). Among the most prevalent vestibular disorders encountered in specialized dizziness clinics are benign paroxysmal positional vertigo (BPPV), vestibular migraine (VM), and persistent postural‐perceptual dizziness (PPPD), which together account for a substantial proportion of patients with chronic or recurrent dizziness (Schifino et al. 2025; Klarendic et al. 2024). Despite their distinct pathophysiological mechanisms, these conditions frequently present with shared comorbidities, including anxiety, depression, and sleep disturbances (Mutlu and Topcu 2022; Eckhardt‐Henn et al. 2008).

Evidence indicates that vestibular input extends beyond brainstem reflex pathways, with widespread connections to central nervous system regions involved in cognition, behaviour, autonomic regulation, and, more recently, sleep physiology (Ried and Arrau 2025). Although the pathophysiology linking sleep and vestibular system remains poorly understood, it has been proposed that sensory organs, including the inner ear, send signals to the Ascending Reticular Activating System through adrenergic pathways, helping regulate attention and wakefulness (Schwarz and Luo 2015). In addition, sleep disturbances, both insufficient sleep and excessive sleep, can exacerbate dizziness symptoms, particularly when the vestibular system is dysfunctional (Smith and Darlington 2013; Mutlu and Topcu 2022). The underlying mechanisms seem to be diverse, reflecting bidirectional cause‐and‐effect relationships that vary across subtypes of sleep and vestibular disorders. However, little is known whether  dizziness is a cause or a consequence of sleep disorders, or vice versa. Indeed, most studies examine dizziness and sleep disorders in broad terms. Given that both encompass a heterogeneous spectrum of conditions, it seems prudent to investigate specific types of sleep disturbances and their associations with distinct dizziness disorders.

Objective assessment of sleep in ambulatory settings has advanced considerably with the development of validated wearable devices. Actigraphy allows for continuous, longitudinal monitoring of sleep‐wake patterns and circadian rhythms under real‐life conditions, while portable home sleep apnoea testing devices (such as the Watch PAT) enable the detection of sleep disordered breathing without the constraints of laboratory based polysomnography (Gonçalves et al. 2015; Ting et al. 2018). The combined use of these tools alongside validated psychometric questionnaires offers a practical approach to sleep characterization that is both ecologically valid and clinically feasible. The present study investigated sleep profiles in patients with PPPD, VM, and BPPV compared to healthy controls, using actigraphy, the Watch PAT portable sleep apnoea testing device, and validated sleep questionnaires, with the hypothesis that each disorder would present a distinct sleep signature reflecting its underlying pathophysiology.

## 2 Materials and Methods

### 2.1 Subjects

We conducted a cross‐sectional study with prospective data collection. The study received ethical approval from the local Ethics in Clinical Research Committee (Hospital Universitário Gaffrée e Guinle/HUGG/UNIRIO; approval number: 64047822.0.0000.5258; approved in November 2022). All patients were diagnosed according to the currently accepted international criteria for each condition: BPPV (Von Brevern et al. 2015), VM (Lempert et al. 2012) PPPD (Staab et al. 2017). Patients were recruited from the Dizziness Clinic of Hospital Universitário Gaffrée e Guinle (HUGG/UNIRIO) between February 2023 and January 2026. All consecutive patients who met the inclusion criteria and none of the exclusion criteria during this period were invited to participate. Healthy controls were recruited from hospital staff and patients’ family members and were required to have no history of vestibular, sleep, or psychiatric disorders at the time of enrolment.

Exclusion criteria included individuals younger than 18 years, conditions impairing walking ability, use of hypnotic, anxiolytic, benzodiazepine, or any medications with direct or indirect sedative effects within 7 days prior to the examination, cognitive impairment that could interfere with study procedures, pregnancy, breastfeeding, or postpartum women at the time of assessment.

Vestibular diagnoses were established by an otolaryngologist and dizziness specialist at the Hospital Universitário Gaffrée e Guinle (HUGG/UNIRIO) according to the diagnostic criteria of the Barany Society (Von Brevern et al. 2015; Staab et al. 2017; Lempert et al. 2012). Patients with overlapping diagnoses among the three study conditions (BPPV, VM, and PPPD), for example, concurrent VM and PPPD were excluded. In patients with BPPV, actigraphy and Watch‐PAT recordings were performed only after the canalith repositioning maneuver had been successfully carried out to treat the acute episode; therefore, sleep was assessed during the post‐treatment period rather than during active positional vertigo.

### 2.2 Procedures

All eligible patients completed self‐assessment questionnaires and subsequently used two wearable devices (placed on the non‐dominant wrist) in sequence: an actigraphy, worn continuously (24 h a day) for 2 weeks, followed by the Watch PAT (a portable home sleep apnoea testing device), used for a single night. Additionally, participants were asked to report their usual get‐up time on weekdays and on weekends, allowing for the calculation of the “weekend–weekday get‐up time”.

### 2.3 DHI

The dizziness handicap inventory (DHI) subjectively measures the functional, physical, and emotional impact of dizziness. The scale identifies three types of difficulty associated with dizziness: functional, emotional, and physical difficulties. It consists of 25 self‐assessment questions, with total scores ranging from 16–34 points (mild handicap), 36–52 points (moderate handicap), to >54 points (severe handicap) (Jacobson and Newman 1990).

### 2.4 PSQI

The Pittsburgh Sleep Quality Index (PSQI) is a questionnaire that assesses sleep quality and disorders over a period of 1 month. It differentiates between “bad” and “good” sleep quality by measuring seven areas (components): subjective sleep quality, sleep latency, sleep duration, habitual sleep efficiency, sleep disturbances, use of sleep medications, and daytime dysfunction in the last month. A total score of 5 or more is indicative of poor sleep quality (Buysse et al. 1989).

Additionally, participants were asked to report their usual get‐up time on weekdays and on weekends, allowing for the calculation of the “weekend–weekday get‐up time”.

### 2.5 BAI and BDI

The Beck anxiety inventory (BAI) is a 21‐item self‐report questionnaire using a Likert scale ranging from 0 to 3, yielding total scores between 0 and 63, with higher scores indicating greater anxiety severity. The Beck depression inventory (BDI) is a 21‐item self‐report instrument designed to assess the presence and severity of depressive symptoms, with scores ranging from 0 to 63, where higher scores reflect greater depressive burden (Richter et al. 1998; Benowitz 2025).

### 2.6 Actigraphy

An actigraph is a device that records activity, using tri‐axial accelerometery data and computational algorithms to differentiate between sleep and wakefulness. In addition, the ambient temperature and exposure to light of the user are also recorded. As such, it gives information such as rest time (surrogate measure of sleep), wake time (surrogate measure of activity), arousals, transition patterns between rest and activity, start and end time of rest, and rest and wakefulness period each day. Participants were instructed to remove the device only during bathing and to press a button to record the time they went to bed and the time they got out of bed; this button did not control the device (i.e., it was not used to turn the device on or off) but served solely to mark these events.

Participants in all groups were instructed to write their sleep‐related information (such as bedtime, wake‐up time, and naps) through a structured daily log over the 14‐day study period. They were also asked to document any relevant events, including removal of the device from the wrist or other occurrences. These self‐reported data were used to support the interpretation of actigraphy results and to minimize potential measurement errors.

A Condor actigraph, model Act Trust, SP (Brazil) was used in this study to determine the robustness of the sleep circadian rhythm. The following variables were assessed: total sleep time (minutes); total time in bed sleep (minutes); sleep efficiency (percentage of time spent asleep while in bed during the night); sleep latency (minutes); sleep onset latency (minutes); WASO (wake after sleep onset, minutes); number of awakenings; the most active period, as indicated by the average activity levels (non‐parametric measure) during the 10 consecutive hours (M10) of highest activity within 24 h; the least active period, as indicated by the average activity levels (non‐parametric measure) during the 5 consecutive hours (L5) of the lowest activity within the 24 h period (Gonçalves et al. 2014; Gonçalves et al. 2015).

### 2.7 Watch PAT

The Watch PAT (WP) system is a home sleep apnoea testing device consisting of a wrist‐worn unit and a finger probe that records peripheral arterial tone (PAT), arterial oxygen saturation, heart rate, and actigraphy, along with snoring and body position via a chest sensor. The device analyses these signals to identify sleep and wake states, sleep stages, and respiratory events, including apnoea, hypopnea, and respiratory effort related arousals. Respiratory events are detected based on PAT amplitude reductions associated with sympathetic activation, defined by one of the following: pulse rate acceleration or increased wrist activity; ≥3% oxyhaemoglobin desaturation; or ≥4% oxyhaemoglobin desaturation alone. All analyses were performed automatically using the device algorithm, without manual scoring or operator intervention. In the present study, the following variables derived from the Watch PAT were analysed: the apnoea‐hypopnea index (AHI), defined as the number of apnoea and hypopnea events per hour of sleep; the oxygen desaturation index (ODI), defined as the number of oxygen desaturation events per hour of sleep; and mean heart rate during sleep (Ting et al. 2018).

It is worth noting that although polysomnography is considered the gold standard for sleep assessment, home‐based methods were preferred in this study for three reasons: to avoid selection bias associated with laboratory sleep studies, particularly given the inclusion of patients with PPPD who may be less likely to accept overnight testing outside their home environment; to ensure greater ecological validity by evaluating sleep under real‐life conditions; and because the American Academy of Sleep Medicine recognizes both actigraphy and Watch PAT as validated tools for assessing circadian rhythm disorders and sleep apnoea, respectively (Smith et al. 2018; Choi et al. 2010).

### 2.8 Statistical Analyses

All analyses were performed using SPSS software, version 26. Given the non‐normal distribution of the data (assessed via Shapiro‐Wilk and visual inspection), variables were log‐transformed prior to parametric testing (ANCOVA) to satisfy model assumptions. The analysis comprised the following methods: comparisons of numerical variables (age and BMI only) among the four groups were performed using one‐way analysis of variance (one‐way ANOVA), followed by Tukey's multiple comparisons test, at a significance level of 5%, to identify statistically significant pairwise differences. For the remaining numerical variables, comparisons among the four groups were conducted using analysis of covariance (ANCOVA), followed by Sidak's multiple comparisons test. As presented in Table 2, the ANCOVA models were adjusted for age only, whereas the models presented in Table 3 were adjusted for both age and BMI; and comparisons of categorical variables among the four groups were carried out using the chi‐square (χ^2^) test or Fisher's exact test, as appropriate. When a significant result was observed, pairwise multiple comparisons were performed using adjusted tests at a significance level of 0.8% to control for Type I error.

The primary variables of interest included: (1) Actigraphy variables: total time in bed, total sleep time, sleep efficiency, onset sleep latency, WASO, number of awakenings, M10, L5; (2) Sleep‐disordered breathing variables measured by Watch PAT: AHI, ODI, heart rate; (3) PSQI total score. The secondary variables were “weekend‐week get up time difference”, BAI, BDI and DHI scores.

## 3 Results

Demographic Characteristics (Table 1): 107 individuals were recruited between February 2023 and January 2026. Twelve patients initially declined to participate, and five withdrew due to discomfort associated with wearing the home sleep device test. A total of 90 participants were included in the final analyses: 24 healthy controls, 21 with BPPV, 23 with PPPD, and 22 with VM. A significant difference in age was observed (*p* = 0.002), with BPPV patients being older than VM patients. BMI also differed significantly (*p* = 0.025), with the BPPV group presenting higher values than controls. No significant difference was found in sex distribution across groups (*p* = 0.44).

**TABLE 1:** Demographic variables across the 4 dizziness groups.

	Control (*n* = 24)	BPPV (*n* = 21)	PPPD (*n* = 23)	VM (*n* = 22)	*p* value	Sig. differences
Age (years)	44 (37–59)	61 (51–68)	59 (41–68)	44 (30–51)	**0.002**	BPPV > VM
BMI (kg/m^2^)	24.5 (21–27)	28.8 (27–33)	25.4 (23–29)	25.8 (24–29)	**0.025**	Control < BPPV
Male/female (*n*)	7/17	2/19	5/18	4/18	0.44	

Age and BMI are expressed as median (Q1–Q3), and Male/Female in absolute frequency (*n*). Inferential analysis was performed for Age and BMI using log‐transformed (LN) data, applying one‐way ANOVA followed by Tukey's multiple comparisons test (α = 5%) to identify significant differences. For male/female the chi‐square (χ^2^) test was used. Legend: BMI = body mass index; PPPD = persistent postural‐perceptual dizziness, BPPV = benign paroxysmal positional vertigo, VM = vestibular migraine.

### 3.1 Actigraphy

Table 2 summarises the results from the Actigraphy across the 4 dizziness groups. Regarding total time in bed, PPPD patients spent significantly more time in bed than VM patients (526 vs. 463 min, *p* = 0.006, Figure 1). Total sleep time was also longest in the PPPD group and shortest in the VM group, with significant differences observed between BPPV and PPPD, and between PPPD and VM (*p* = 0.003). Sleep onset latency did not differ significantly across groups (*p* = 0.58).

**TABLE 2:** Comparison of the sleep variables across groups (adjusted for age).

	**Control (*n* = 24)**	**BPPV (*n* = 21)**	**PPPD (*n* = 23)**	**VM (*n* = 22)**	***p* value**	**Sig. differences**
**Objective variables measured using actimetry**						
Total time in bed (min)	480 (452–524)	466 (443–532)	526 (464–583)	463 (416–510)	**0.006**	PPPD > VM
Total sleep time (min)	411 (380–448)	383 (333–398)	423 (382–505)	370 (338–420)	**0.003**	PPPD > BPPV, PPPD > VM
Onset sleep latency (min)	9.5 (7–13)	7.0 (4–16)	10.0 (7–15)	11.0 (7–18)	0.58	
WASO (min)	50.5 (35–72)	90 (56–123)	60 (44–71)	69 (51–112)	**0.003**	BPPV > Control, BPPV > PPPD
Sleep efficiency (%)	86.5 (83–90)	78 (71–84)	87 (82–88)	82.9 (75–86)	**0.015**	BPPV < Control, BPPV < PPPD
Number of awakenings	7.8 (6.7–10)	11.0 (8–13)	9.7 (5.8–11)	10.7 (8–14)	**0.008**	BPPV > Control, BPPV > PPPD
M10	3408 (2779–3808)	2913 (2766–3796)	2701 (2125–3376)	4114 (3059–4983)	**0.012**	PPPD < VM
L5	155 (126–331)	276 (156–415)	149 (103–272)	215 (164–304)	**0.036**	BPPV > PPPD
**Subjective variables derived from questionnaires**						
PSQI total score	5.0 (4–8)	8.0 (6–10)	11.0 (9–14)	9.0 (8–13)	**<0.0001**	Control < BPPV, Control < PPPD, Control < VM
Weekend–week get up time difference (min)	60 (7.5–120)	80 (0–120)	0 (0–60)	120 (33.8–180)	0.072	
DHI	—	42 (27–61)	64 (48–72)	33 (20–59)	**0.015**	PPPD > VM
Beck depression	5.5 (3–10)	10 (6.5–12)	19 (11.0–23)	11.5 (7–15)	**<0.0001**	Control < PPPD, Control < VM
Beck anxiety	3.5 (1.3–10.8)	11 (7.5–20)	20 (15.0–28)	12 (7.0–27)	**<0.0001**	Control < BPPV, Control < PPPD, Control < VM

*Note*: Variables are expressed as median (Q1–Q3). Inferential analysis was performed using log‐transformed (LN) data, applying ANCOVA adjusted for age followed by Sidak's multiple comparisons test (α = 5%) to identify significant differences. “*p* value” corresponds to the overall ANCOVA. Sleep efficiency is the percentage of time spent asleep while in bed during the night. Legend: PPPD = persistent postural‐perceptual dizziness, BPPV = benign paroxysmal positional vertigo; VM = vestibular migraine; WASO = Wake after Sleep Onset; M10 is the average activity levels (non‐parametric measure) during the 10 consecutive hours (M10) of highest activity within 24 h; L5 is the average activity levels (non‐parametric measure) during the 5 consecutive hours (L5) of the lowest activity within 24 h; PSQI = Pittsburgh sleep quality index; DHI = dizziness handicap inventory.

BPPV patients showed the highest WASO values (90 min), significantly greater than both controls (50.5 min) and PPPD patients (60 min) (*p* = 0.003). Consistently, sleep efficiency was lowest in the BPPV group (78%), significantly lower than controls (86.5%) and PPPD patients (87%) (*p* = 0.015, Figure 2). The number of nocturnal awakenings followed the same pattern, being highest in BPPV (11.0), with significant differences against controls (7.8) and PPPD (9.7) (*p* = 0.008).

With respect to circadian activity patterns, M10 (reflecting daytime activity levels) was significantly lower in PPPD (2701) compared to VM (4114) (*p* = 0.012), suggesting reduced daytime mobility in PPPD patients. L5 (reflecting nocturnal activity) was highest in BPPV (276) and significantly greater than in PPPD (149) (*p* = 0.036), consistent with greater nocturnal restlessness in the BPPV group.

### 3.2 WatchPAT

AHI was markedly elevated in the BPPV group (31.5 events/hour) compared to all other groups (Figure 3), with a statistically significant difference against VM (5.2 events/hour) (*p* = 0.003). The oxygen desaturation index (ODI) followed the same pattern, being highest in BPPV (Lempert et al. 2012) and significantly greater than in VM (2.3) (*p* = 0.009). Heart rate during sleep was significantly higher in BPPV (69 bpm: IQR 65–79) compared to both controls (62 bpm: IQR 55–66) and PPPD (63 bpm: IQR 56–69) (*p* = 0.002).

### 3.3 Questionnaires

The PSQI total score differed significantly across all groups (*p* < 0.0001, Figure 4). All three dizziness groups reported worse subjective sleep quality than controls, with PPPD patients presenting the highest scores (11.0), followed by VM (9.0) and BPPV (8.0), compared to controls (5.0).

BAI scores were significantly higher in all dizziness groups compared to controls (*p* < 0.0001), with PPPD showing the greatest anxiety burden (20.0), followed by VM (12.0) and BPPV (11.0). Similarly, BDI scores were significantly higher in PPPD and VM groups compared to controls (*p* < 0.0001), with PPPD again presenting the highest values (19.0).

The DHI was significantly higher in PPPD (64; IQR: 48–72) compared to VM (33; IQR: 20–59) (*p* = 0.015), reflecting greater dizziness‐related disability in PPPD patients.

The weekend–weekday get‐up time difference did not reach statistical significance across groups (*p* = 0.072); however, a notable pattern was observed, with VM patients showing the greatest discrepancy (120 min; IQR: 33.8–180) and PPPD patients showing virtually none (0 min; IQR: 0–60).

## 4 Discussion

The present study aimed to compare sleep profiles across the commonest vestibular disorders (BPPV, PPPD, and VM) and healthy controls, using a comprehensive multimodal approach combining actigraphy, home sleep apnoea testing (Watch PAT), and PSQI. While all three dizziness conditions shared a common burden of poorer sleep quality compared to healthy controls, each disorder presented distinct sleep markers reflecting its underlying pathophysiology.

### 4.1 Common Psychological and Sleep Burden Across Vestibular Disorders

A consistent finding across all dizziness groups was the significantly worse subjective sleep quality on the PSQI, alongside higher scores on the BAI and BDI, compared to healthy controls (Table 2; Figure 4). This pattern is consistent with previously reported findings (Wang et al. 2018; Mei et al. 2020; Mutlu and Topcu 2022; Wu et al. 2020) and supports the notion that sleep, anxiety, depression, and dizziness are mutually reinforcing conditions, rather than a simple cause‐and‐effect sequence. For clinicians managing patients with dizziness, this has an important practical implication: sleep quality and psychological burden should be routinely assessed as part of the clinical evaluation, regardless of the specific vestibular diagnosis.

### 4.2 Sleep Fragmentation and Sleep‐disordered Breathing

We observed a markedly elevated AHI and ODI in the BPPV group (Table 3; Figure 3), which were significantly higher than those recorded in the VM group. Consistent with this, actigraphy revealed that BPPV patients spent significantly more time awake after sleep onset, had lower sleep efficiency (Table 2 and Figure 2), and experienced more nocturnal awakenings than both controls and PPPD patients. These findings suggest that sleep apnoea was the main cause of poor sleep in the BPPV group. Notably, these differences remained robust even after adjustment for BMI. A higher BMI was observed in our BPPV group, which was unexpected. Although the elevated AHI and ODI in the BPPV group remained significant after ANCOVA adjustment for age and BMI, residual confounding cannot be entirely excluded. There is some evidence that hyperinsulinemia and hyperglycaemia are risk factors for BPPV (Webster et al. 2015), raising the question of whether BPPV may be associated with broader metabolic dysfunction—a hypothesis that merits investigation in future studies.

**TABLE 3:** Comparison of the WatchPAT variables across groups (adjusted for age and BMI).

	Control (*n* = 24)	BPPV (*n* = 21)	PPPD (*n* = 23)	VM (*n* = 22)	*p* value	Sig. differences
AHI	11.8 (6.5–23)	31.5 (19.2–50)	18.3 (7.4–29)	5.2 (2.4–13)	**0.003**	BPPV > VM
ODI	3.8 (1.4–6.1)	16 (6.9–25)	7.3 (2.7–20)	2.3 (0.25–4.7)	**0.009**	BPPV > VM
HR	62 (55–66)	69 (65–79)	63 (56–69)	69 (61–77)	**0.002**	BPPV > Control, BPPV > PPPD

*Note*: Variables are expressed as median (Q1–Q3). Inferential analysis was performed using log‐transformed (LN) data, applying ANCOVA adjusted for age and BMI followed by Sidak's multiple comparisons test (α = 5%) to identify significant differences. “*p* value” corresponds to the overall ANCOVA. Legend: BMI = body mass index, PPPD = persistent postural‐perceptual dizziness, BPPV = benign paroxysmal positional vertigo, VM = vestibular migraine, AHI = apnea hypopnea index, ODI = oxygen desaturation index, HR = heart rate.

OSA is characterized by recurrent upper airway obstruction during sleep (leading to intermittent hypoxemia, autonomic dysregulation, and sleep fragmentation) and carries significant systemic burden, with long‐term consequences: hypertension, coronary artery disease, arrhythmias, cerebrovascular disease, cognitive decline, and type 2 diabetes (Yeghiazarians et al. 2021). Given that the labyrinth are supplied by a terminal artery without collateral circulation, they are particularly vulnerable to the endothelial damage caused by intermittent hypoxemia (Yu et al. 2025). It makes them particularly vulnerable to otolith detachment and may potentially contribute to the development of BPPV (Gallina et al. 2010). Beyond hypoxemia, autonomic dysregulation (reflected by altered heart rate variability) has also been associated with OSA (Lin et al. 2023). Although the Watch PAT does not provide heart rate variability data, the higher mean heart rate during sleep observed in the BPPV group (Table 3) may serve as an indirect marker of this autonomic burden, consistent with the greater sleep apnoea load observed in this patient population.

Interestingly, despite the tendency of BPPV patients to limit head movement to avoid triggering vertiginous episodes (a behaviour that might be expected to reduce nocturnal activity), the highest nocturnal movement (L5) was paradoxically observed in this group (Table 2). Rather than reflecting voluntary movement, we attribute this to involuntary restlessness driven by the frequent arousals associated with sleep apnoea events, further reinforcing sleep apnoea as the principal disruptor of sleep in this population, although we do not have polysomnography data to confirm this.

### 4.3 Sleeping More, Moving Less, Feeling Worse: The PPPD Sleep Paradox

Although the PPPD group presented the longest total sleep time and highest sleep efficiency (Table 2) their subjective perception of sleep quality (PSQI) was the poorest among all groups (Figure 4; Table 2). This discrepancy between objective and subjective sleep measures is a well‐recognized pattern in functional disorders, where negative perception tends to be disproportionately greater than objective findings (San Pedro Murillo et al. 2022). It is also consistent with paradoxical insomnia, a condition in which patients perceive their sleep as significantly worse than what is objectively recorded, in the absence of measurable sleep disturbance (Edinger and Krystal 2003).

PPPD patients spent more time in bed than all other groups (Table 2, Figure 1), with a statistically significant difference against the VM group. This is likely because PPPD patients feel less dizzy when lying down than when standing (a hallmark feature of the condition) which naturally encourages them to stay in bed longer. It is important to note that increased time in bed, combined with poor subjective sleep perception, high anxiety levels, and physical inactivity, are well‐recognized factors to develop insomnia over time (Spielman et al. 1987). Therefore, despite their preserved sleep efficiency, PPPD patients carry several risk factors for insomnia in the long term.

**FIGURE 1:**
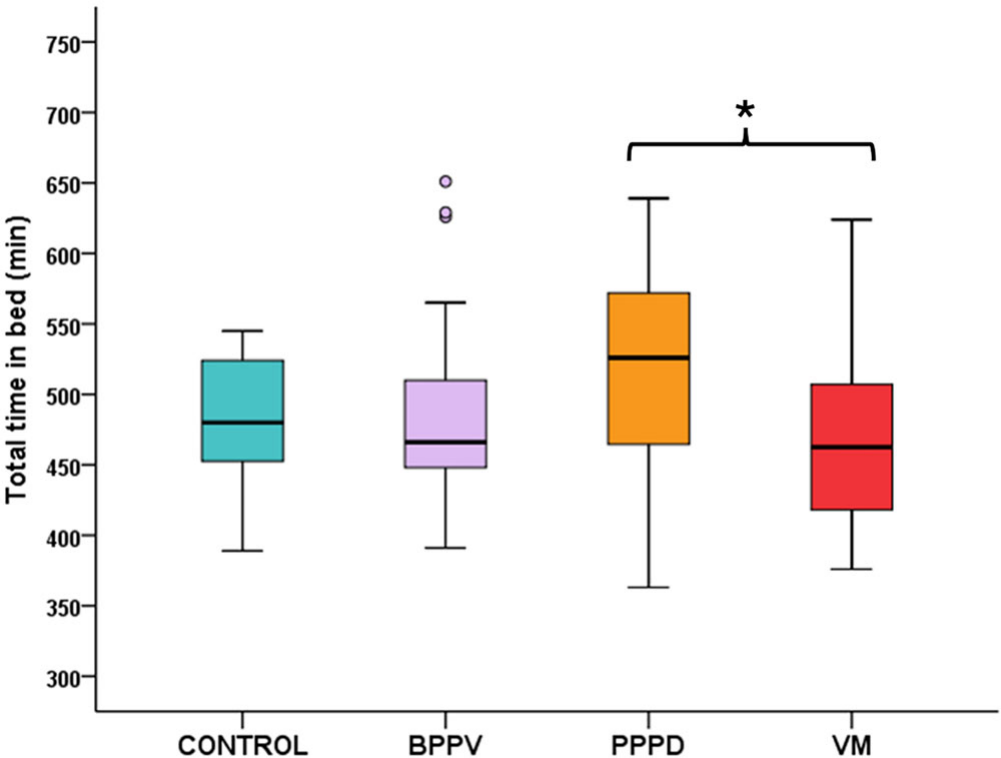
Boxplot of “Total time in bed” across group. *Note*: Data are presented as median with interquartile range. Brackets with * indicate statistically significant differences between groups as identified by Sidak's multiple comparisons test (*p* < 0.05). Legend: PPPD = persistent postural‐perceptual dizziness, BPPV = benign paroxysmal positional vertigo, VM = vestibular migraine.

**FIGURE 2:**
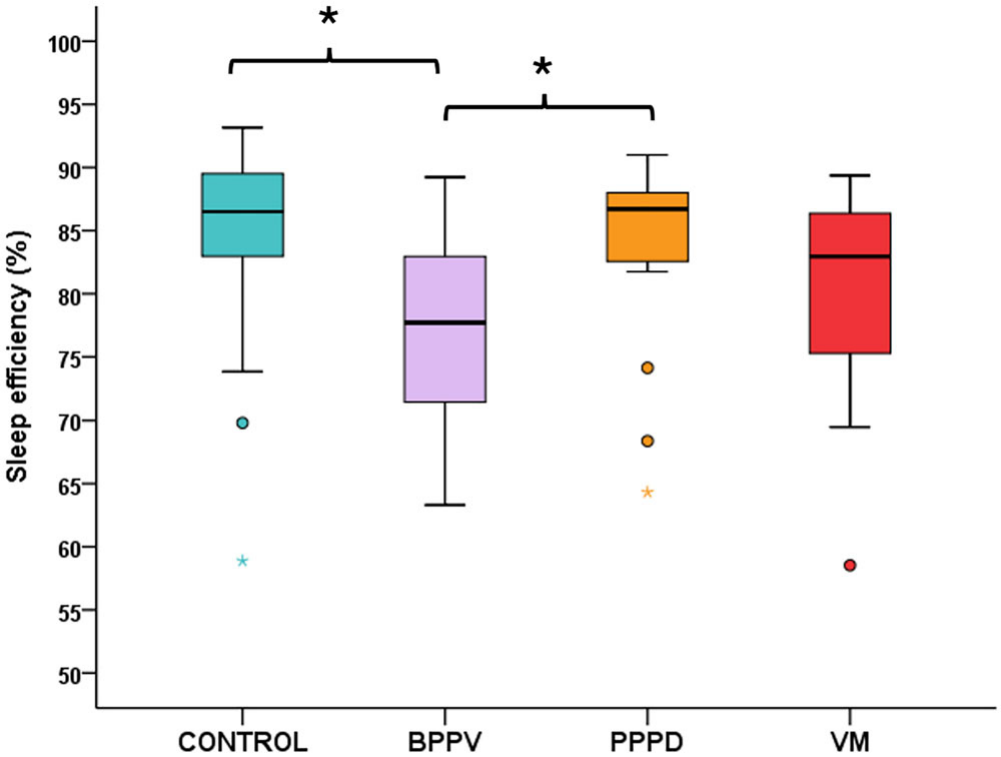
Boxplot of “Sleep Efficiency” across group. *Note*: Sleep efficiency is the percentage of time spent asleep while in bed during the night. Brackets with * indicate statistically significant differences between groups as identified by Sidak's multiple comparisons test (*p* < 0.05). Data are presented as median with interquartile range. Legend: PPPD = persistent postural‐perceptual dizziness, BPPV = benign paroxysmal positional vertigo, VM = vestibular migraine.

This picture of reduced activity in PPPD extends beyond the bedroom. During the day, PPPD patients showed the lowest activity levels (M10) of all groups, significantly lower than VM patients, likely driven by fear of falling and heightened sensitivity to motion (Kaski 2024). Our group has previously suggested that chronic physical immobility in patients with persistent dizziness promotes social isolation, worse sleep quality, and may play a role in differentiating PPPD from VM (Joffily et al. 2025). In addition, at night, activity assessed by the L5 variable indicated that PPPD patients exhibited lower movement compared to all other groups, with a significant difference observed in relation to BPPV. This finding suggests that individuals with PPPD maintain a protective behavioural even during sleep. In addition, time‐in‐bed restriction therapy is one of the cornerstones of Cognitive Behavioural Therapy for Insomnia (Edinger and Krystal 2003), which contrasts with the maladaptive compensatory behaviour observed in patients with PPPD.

### 4.4 Chronic Sleep Deprivation and Social Jetlag

Some authors examined sleep patterns in a large sample of migraine patients and identified sleep deprivation as one of the most reported triggers for migraine attacks (Kelman and Rains 2005). In our study, total sleep time was shorter in the VM group than in other groups (Table 2). Although the difference in get‐up time between weekdays and weekends did not reach statistical significance across groups (*p* = 0.072), a clinically meaningful divergent pattern emerged between VM and PPPD patients (Table 2, Figure 5). A larger sample would provide greater statistical power to determine whether this pattern reflects a true group difference.

**FIGURE 3:**
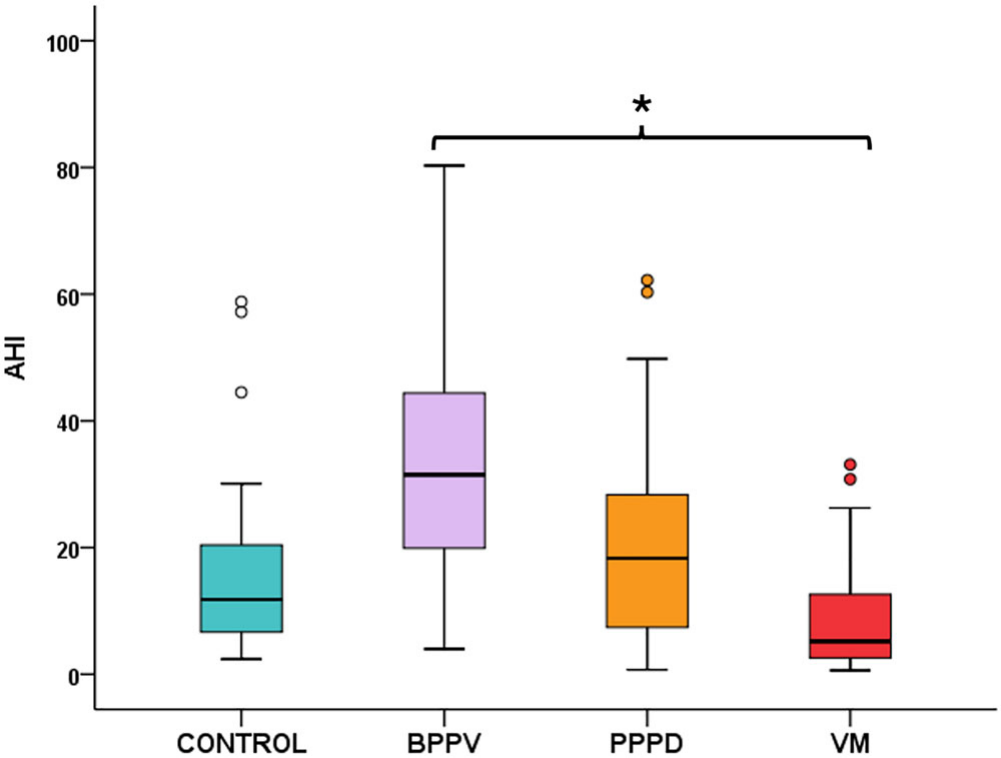
Boxplot of AHI across groups. *Note*: AHI (Apnea Hypopnea Index) represented by number of apnea and hypopnea events per hour of sleep. Data are presented as median with interquartile range. Brackets with * indicate statistically significant differences between groups as identified by Sidak's multiple comparisons test (*p* < 0.05). Legend: PPPD = persistent postural‐perceptual dizziness, BPPV = benign paroxysmal positional vertigo, VM = vestibular migraine.

**FIGURE 4:**
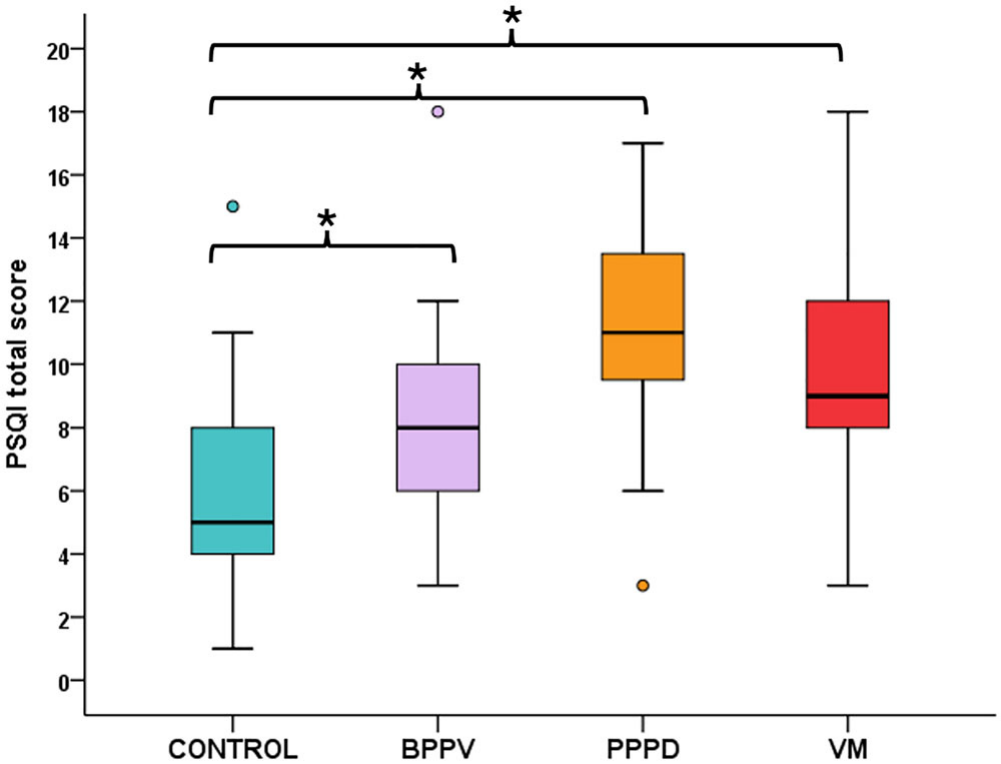
Boxplot of PSQI across groups. *Note*: Data are presented as median with interquartile range. Brackets with * indicate statistically significant differences between groups as identified by Sidak's multiple comparisons test (*p* < 0.05). Legend: PSQI = Pittsburgh sleep quality index, PPPD = persistent postural‐perceptual dizziness, BPPV = benign paroxysmal positional vertigo, VM = vestibular migraine.

**FIGURE 5:**
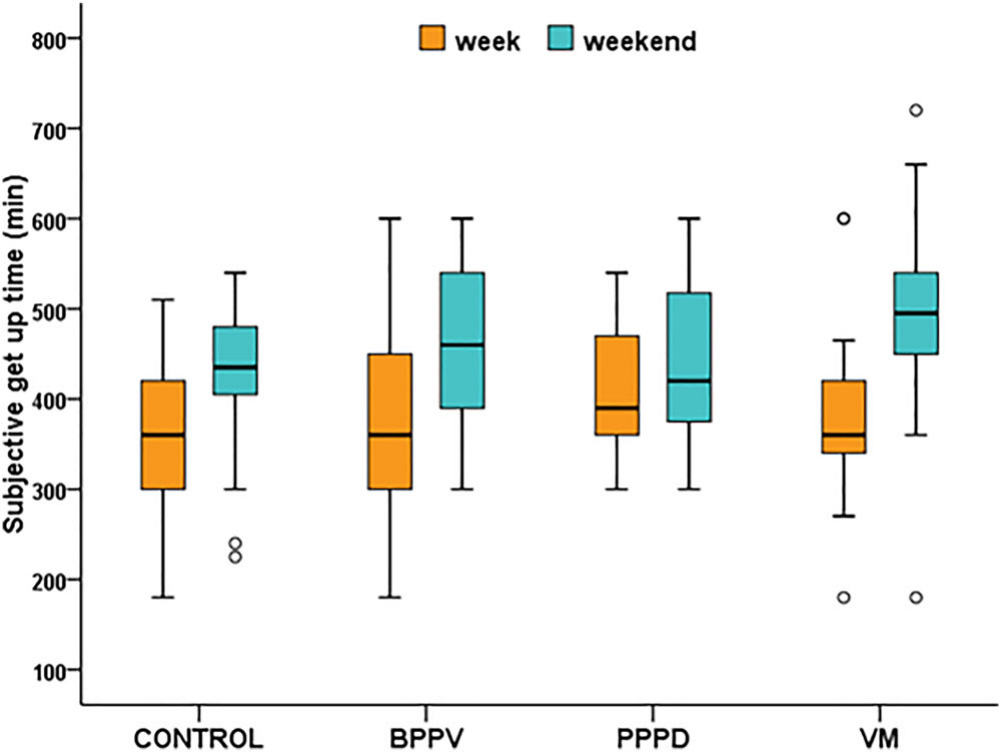
Boxplot of subjective “Get up time week and weekend” across groups. *Note*: Data are presented as median with interquartile range. Legend: PPPD = persistent postural‐perceptual dizziness, BPPV = benign paroxysmal positional vertigo, VM = vestibular migraine.

The PPPD group was the only one that showed virtually no difference between weekday and weekend get‐up times, likely reflecting the behavioural rigidity commonly observed in these patients, who rely on strict daily routines to manage anxiety and avoid the distress caused by disruptions to their schedule. In contrast, the VM group showed the greatest discrepancy, suggesting a tendency to extend sleep on free days to compensate for accumulated weekday sleep debt, pointing to an unmet sleep need that is systematically constrained by social and occupational demands.

Those latter finding suggests that the VM group may be more chronically sleep‐deprived, particularly when compared to the PPPD group. This pattern of extending sleep on free days to recover from accumulated weekday sleep debt is the behavioural signature of social jetlag, a state of chronic circadian misalignment arising when the social clock, the solar clock, and the biological clock fall out of sync. Moreover, social jetlag has been associated cyclic adverse health outcomes, including metabolic dysfunction, depressive symptoms, and poorer lifestyle habits (Wittmann et al. 2006; Roenneberg et al. 2019). Beyond fatigue, chronic circadian misalignment carries neuroinflammatory implications that may act as direct triggers for migraine attacks, creating a self‐perpetuating cycle in which por sleep provokes migraines, and migraines in turn further disrupt sleep (Vgontzas and Pavlović 2018).

These findings suggest hypothesis for clinical practice: since each vestibular disorder presents a distinct sleep profile (Figure 6), sleep assessment and management should be tailored to the specific diagnosis rather than adopting a uniform approach. In BPPV, the high prevalence of sleep‐disordered breathing suggests that screening for Sleep apnoea should be considered, particularly in patients with recurrent episodes. Although direct evidence that CPAP therapy reduces BPPV recurrence remains scarce, it is reasonable to hypothesize that treating sleep apnoea could reduce sleep fragmentation, and thereby potentially lower the risk of recurrence. In PPPD, given that cognitive behavioural therapy is the gold standard treatment for both chronic insomnia (Edinger and Krystal 2003) and PPPD (Staab et al. 2017), its application in patients with comorbid PPPD and sleep complains represents a particularly promising therapeutic approach, with the potential to simultaneously address both conditions. In VM, addressing chronic sleep insufficiency and encouraging regular sleep schedules could represent a helpful management strategy (Calhoun and Ford 2007). Overall, the interventions discussed above should be regarded as hypotheses or potential clinical implications rather than evidence‐based therapeutic recommendations, pending confirmation in dedicated prospective interventional studies.

**FIGURE 6:**
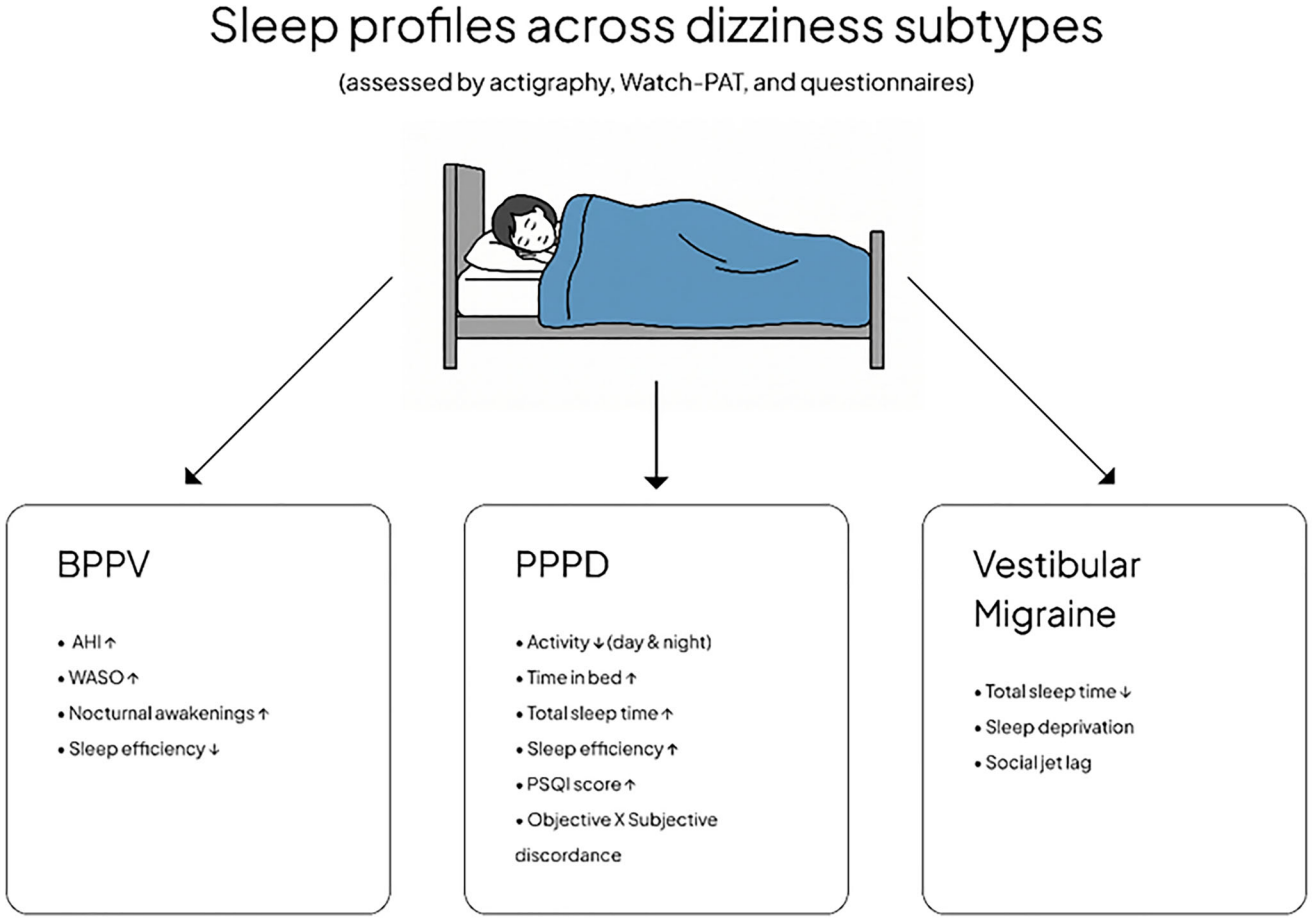
Sleep profiles across dizziness subtypes assessed by actigraphy, Watch‐PAT, and questionnaires. *Note*: Sleep efficiency is the percentage of time spent asleep while in bed during the night. Legend: PPPD = persistent postural‐perceptual dizziness, BPPV = benign paroxysmal positional vertigo; VM = vestibular migraine; AHI = Apnea Hipopnea Index; WASO = Wake after Sleep Onset; PSQI = Pittsburgh Sleep Quality Index. Arrows indicate the direction of change (↑ increase, ↓ decrease) relative to controls.

We acknowledge certain limitations of the present study. First, the absence of complementary polysomnography that may have constrained the depth of sleep architecture assessment. Second, the relatively small sample size across four groups may have limited statistical power for between group comparisons. Non‐significant findings, such as the weekend–weekday get‐up time difference, should therefore be interpreted with caution, as a larger, adequately powered sample could potentially reveal differences that did not reach significance in the present study. Third, the study did not account for shift work status, working days and hours (which may influence sleep routines), nor did it consider individual chronotype (‘morningness’ & ‘eveningness’ preferences), which could have provided additional context for the circadian findings observed.

## 5 Conclusion

In summary, across all three conditions, sleep disturbance, anxiety, and depression were universally more pronounced than in dizziness‐free controls, reinforcing the bidirectional relationship between sleep, emotional burden, and chronic dizziness. BPPV were characterized by objective sleep fragmentation driven by a higher sleep apnoea index. PPPD demonstrated reduced activity levels (day and night) and a paradoxical sleep pattern (higher sleep efficiency with the poorest subjective sleep perception), consistent with the objective‐subjective disconnect characteristic of functional disorders. VM patients showed shorter total sleep time and a non‐significant trend toward a larger weekend–weekday get‐up discrepancy, raising the hypothesis of sleep insufficiency, possibly related to social jetlag, to be tested in larger studies. These findings reinforce that dizziness is not a homogeneous entity, and sleep management should be tailored accordingly in clinical practice.

## Author Contributions

**Lucia Joffily**: conceptualization, data acquisition, data curation, analysis, methodology, validation, visualization, writing – original draft, writing – review and editing. **Marco Antonio Sales Dantas de Lima**: project administration, supervision, methodology, validation, visualization, writing – review and editing. **John Fontenele Araújo**: supervision, validation, writing – review and editing. **Diego Kaski**: methodology, supervision, validation, writing – review and editing. All authors read and approved the final version of the manuscript and agree to be accountable for all aspects of the work.

## Funding

The authors have nothing to report.

## Conflicts of Interest

The authors declare no conflicts of interest.

## Data Availability

De‐identified data supporting the findings of this study are available from the corresponding author upon reasonable request, subject to institutional approvals and applicable data protection regulations.
